# 10-Year Evaluation of the First Root Analogue Implant on Humans, Made Using a CT Scan, CAD/CAM and DMLS

**DOI:** 10.3390/biomimetics7010032

**Published:** 2022-03-09

**Authors:** Michele Mario Figliuzzi, Domenico Aiello, Carlo Rengo, Luca Parentela, Carlo Mangano

**Affiliations:** 1 Department of Health and Oral Sciences Periodontology Clinic, Medicine and Surgery School, “Magna Graecia” University of Catanzaro, 88100 Catanzaro, Italy; d.aiello@unicz.it (D.A.); lucaparentela97@gmail.com (L.P.); 2 Dental School of Periodonotogy, University of Naples “Federico II”, 80127 Napoli, Italy; carlorengo@alice.it; 3 Department of Medicine and Surgery, Dental School, University of Varese, 21100 Varese, Italy; cmangan@gmail.com

**Keywords:** implants, root-analogue, DLMS, titanium, CAD/CAM

## Abstract

Ten years ago, for the first time in humans, thanks to the DLMS (direct metal laser sintering) technique, we designed, built and inserted an immediate post-extraction custom-made root-analogue implant in Ti-6Al-4v with platform switching. The implant was inserted into the post-extraction socket, respecting the biological width. After 10 years, we wanted to evaluate the dimensional stability of the implant and the eventual crestal bone resorption. The evaluation was performed clinically with periodontal parameters and radiographically by means of an intraoral X-ray with the parallel technique measuring the distance between the base of the bone crest and the implant shoulder. It appears that the implant has maintained dimensional stability of the peri-implant soft tissues, and the crestal resorption is 0 mm. This could represent a step forward to make this experimental method a valid alternative to the current immediate post-extraction implant procedures in use.

## 1. Introduction

In the dental field, implant rehabilitation is increasingly in demand. Immediate post-extraction implants are inserted immediately after the avulsion of dental elements [1]. This is certainly a technique that must be performed with caution, as failures, as compared to the insertion of a delayed implant, once the socket is healed, can be more frequent [2]. A fundamental requirement for the success of this procedure is to obtain adequate primary stability; to obtain this, it is necessary for the implant to have the right macroscopic characteristics and that the alveolus is preserved during the surgical dental removal. It has been hypothesized that this could be a valid alternative to common implants when proposing custom-made implants that have a shape similar to that of the extracted root [3]. Direct laser metal sintering (DLMS) [4] is a technique which allows solids with a complex geometry to be produced by annealing metal powder microparticles in a focused laser beam, according to a computer-generated three-dimensional (3D) model. For dental implants, the fabrication process involves the laser-induced fusion of titanium microparticles in order to build, layer by layer, the desired object. In other fields additive manufacturing (AM) is one of the established processes for three-dimensional (3D) printing. Since osseointegration is such an important factor in the success of dental implants, it may be biologically beneficial to use porous implants, extending the features that promote osseointegration beyond the surface, throughout the body of the device [5,6]. With conventional methods (such as plasma spraying), however, it is impossible to fabricate a porous structure with a completely controlled design of the external shape as well as the internal pore network, with the tight constraints of porosity, optimum pore size, and mechanical strength that are required. With the DLMS technique, it is possible to control the porosity of each layer, but also pore interconnectivity, size, shape, and distribution, and consequently the 3D architecture of the implant, by changing the processing parameters. This is an important advantage of this technique: a high level of interconnectivity resulting in a predominantly open-pored morphology may allow bone ingrowth and vascularization, thus enhancing osseointegration, the essential factor of the long-term reliability of an implant [7,8,9].

Over 10 years, we wanted to evaluate the dimensional stability of our RAI custom-made implant and the eventual crestal bone resorption with the aim of evaluating the long-term outcomes of the procedure, while also comparing it to standard methods and other studies on long-term RAIs, to take a further step towards the recognition of this new technique to expand the possibilities that clinicians have at their disposal [10,11,12,13].

## 2. Materials and Methods

Ten years ago, with the DLMS technique, a root analogue implant (RAI) in Ti-6Al-4v (given the excellent properties of the material) [14] was produced from three-dimensional models developed through the 3D processing of radiographic images, obtained through CT, of the future post-extraction alveolus [15]. This implant was positioned inside the post-extraction socket, kept intact through an avulsion that was as atraumatic and conservative as possible. The selected patient was a healthy 50-year-old female with a fracture of a maxillary second premolar. The patient’s informed consent was verbally obtained for them to take part in the study. The patient was a woman in good health, not suffering from systemic pathologies, not a smoker, with an average lifestyle. No pathological symptoms of the custom-made implant were declared by the patient over the 10 years, and instead the patient considers the treatment very satisfactory, both from a functional and an aesthetic point of view. Excellent primary stability was achieved, thanks to the perfect correspondence between the walls of the alveolus and the shape given to the custom-made implant, as evaluated by percussion and palpation. A metal-free capsule would not have adhered adequately to the custom-made implant, so we opted for a metal-ceramic restoration. Cementation was carried out with GC Fuji I, a highly effective glass ionomer cement. At the subsequent and numerous follow-ups, the outcome of the procedure was clinically and radiographically assessed and a good adaptation of the peri-implant tissues and excellent osseointegration without any signs or pathological symptoms and without any bone resorption was highlighted.

From Time 0 (T0—when the implant was placed with the method described), methods for performing proper home dental hygiene were described to the patient. Furthermore, in agreement with the patient, a periodic recall system was set, and assiduously respected; after 1, 2, 4, 5, 8 and 10 y, the patient attended observations for routine checks and implant maintenance interventions.

During the checks, the patient was subjected to an anamnestic examination, looking for the possible presence of pathological symptoms. Meticulous clinical examinations were carried out by observation, palpation, percussion and probing, collecting periodontal/peri-implant health index data. Intraoral X-rays were performed with the parallel technique supported by customized resin checks.

GXIO-770 Gendex was used to take the Rx pictures.

## 3. Results

**Figure 1:**
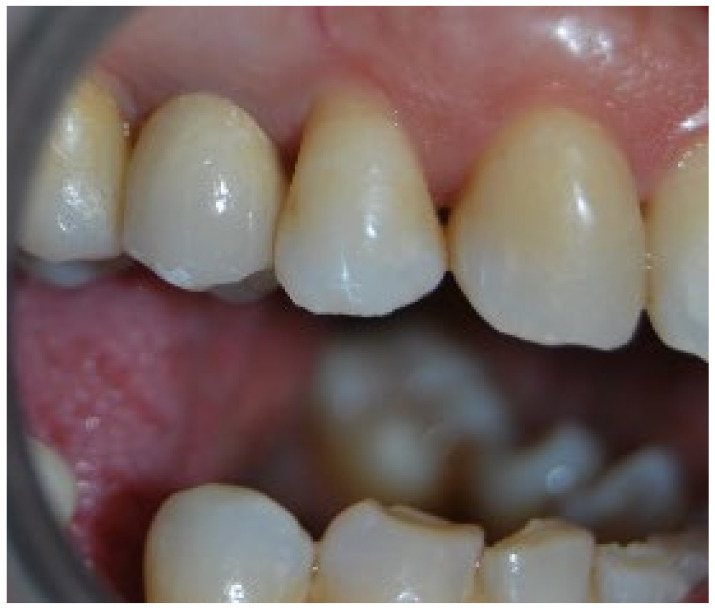
Two-year clinical follow-up. Clinical photo 2 years after implantation.

**Figure 2:**
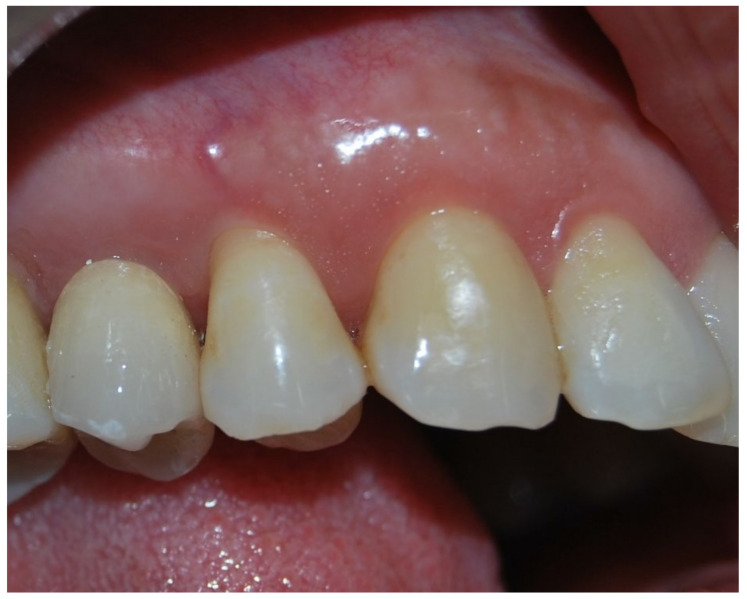
Five-year clinical follow-up. Clinical photo 5 years after implantation.

**Figure 3:**
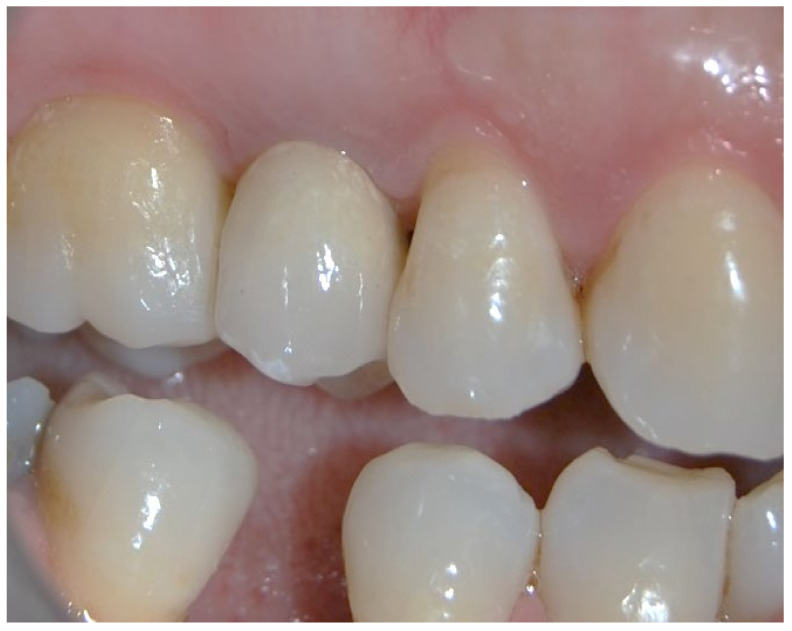
Clinical photo of the patient 10 years after the procedure.

**Figure 4:**
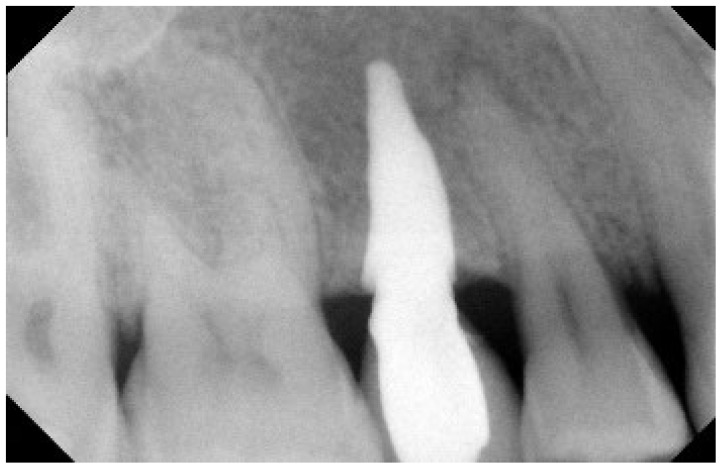
Two-year Rx follow-up. Rx check, 2 years after implantation.

**Figure 5:**
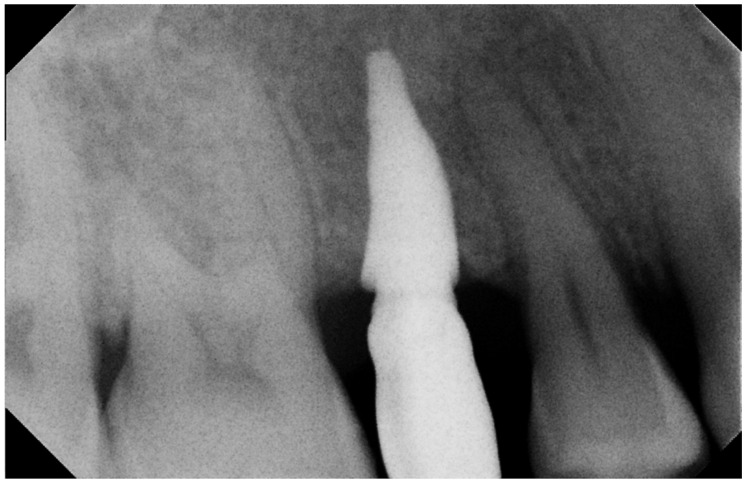
Five-year Rx follow-up. Rx check 5 years after implantation: the prosthetic crown was provisionally cemented to evaluate the clinical course over time (e.g., any infiltrations, etc.), the Rx image at the 5th year was captured after removing the crown to carry out the routine check established in the therapeutic plan, and so it was not present.

**Figure 6:**
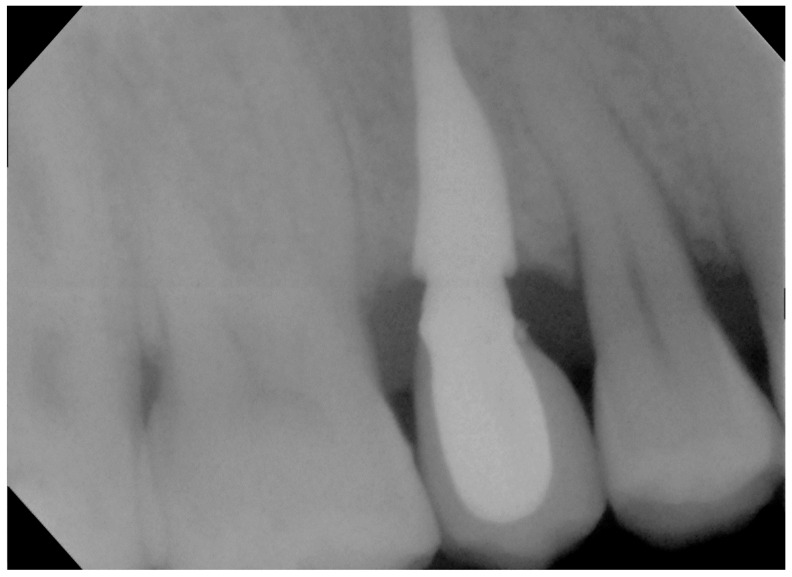
Rx with customized resin check 10 years after the procedure.

No pathological symptoms were reported by the patient. As shown in clinical photographs (Figure 1, Figure 2 and Figure 3), over the years, the peri-implant tissues have adapted perfectly and remain clinically healthy. Probing pocket depth (PPD) was within the normal range of values (2.5 mm), bleeding on probing (BOP) was not present, there was no sign of gingival recession, no increased mobility, and excellent dimensional stability was highlighted. No signs of bone resorption (0 mm) were evident in radiographs (Figure 4, Figure 5 and Figure 6), considering the distance between the base of the bone crest and the implant shoulder as a reference.

## 4. Discussion

The results obtained are very satisfactory, as the crestal bone has not altered its size over time and no complications have been highlighted. This method seems to give predictable results over time, comparable and even better than those obtained with traditional methods [11,12,13] (although we realize that this is a single case and therefore this statement requires further confirmation that we trust will be provided in the near future). The excellent results obtained may be due to the structural advantages in terms of osseointegration of the custom-made implant compared to those made traditionally, to the perfect match of the implant structure with the walls of the socket, to the correct selection of the patient (middle-aged woman in good health, collaborating, without interfering risk factors such as systemic disease), to a good surgical protocol (conservative, with maintenance of the periosteal vascularization and maintenance of the integrity of the alveolar walls) and to the less invasive implant insertion technique that, compared to a traditional implant, it is not screwed into the bone but positioned in place, mimicking the root anatomy of the lost natural tooth. At the present stage, the limits seem to be of an organizational nature, as designing and producing individualized products with this technique requires several design phases. In recent years, various 3D printing methods have spread, and some of these are so common that there are machines produced for hobby use; it is not unthinkable that the DLMS production technique could be made more available in the future. If this turns out to be correct and further studies continue to confirm the effectiveness of these custom-made implants thus produced [16], this technique could be associated with common implant methods.

## Data Availability

The data presented in this study are available on request from the corresponding author as they are part of a patient’s medical record.
